# Solid-State Transformation (*S*_total_ = 0, 1, and 2) in a Ni^2+^ Chelate with Two *tert*-Butyl 5-(*p*-Biphenylyl)-2-pyridyl Nitroxides

**DOI:** 10.3390/ma18122793

**Published:** 2025-06-13

**Authors:** Masataka Mitsui, Takayuki Ishida

**Affiliations:** Department of Engineering Science, The University of Electro-Communications, Chofu 182-8585, Tokyo, Japan

**Keywords:** aminoxyl, heterospin, exchange coupling, spin transition, spin crossover

## Abstract

A novel *S* = 1/2 paramagnetic chelating ligand *tert*-butyl 5-(*p*-biphenylyl)-2-pyridyl nitroxide (bppyNO) and its *S* = 1 nickel(II) ion complex [Ni(bppyNO)_2_Br_2_] were synthesized. X-ray crystallography revealed a 2p–3d–2p heterospin triad, with half of the molecule being crystallographically independent. A relatively planar chelate geometry with the torsion angle *ϕ*(Ni-O-N-C_2py_) = −10.6(5)° at 300 K becomes significantly out-of-plane distorted with *ϕ* = −46.9(8) and 26.1(11)° at 90 K accompanied by disorder at the oxygen site. The torsion angle changes, Δ*ϕ* = 36° and 37°, are among the largest reported for related compounds. Magnetic measurements indicate gradual and incomplete spin transition-like behavior around 143(2) K. A three-state model involving an intermediate-spin (*S*_total_ = 1) state is proposed to explain non-zero *χ*_m_*T* plateau in a low-temperature region. Density functional theory calculations using the determined structures support the proposed mechanism. Furthermore, geometry optimizations assuming *S*_total_ = 0, 1, and 2 are in good agreement with the present model.

## 1. Introduction

The dynamics modulating the ground-state spin of a molecule open up exciting possibilities for the development of switchable functional materials as an aspect of polymorphism or solid-state phase transition, such as spin crossover (SCO) compounds [1] and systems exhibiting SCO-like behavior [2,3,4]. Among these, interconversion between diamagnetic (*S* = 0) and paramagnetic (*S* = 2) states has been most extensively studied in 3d⁶ iron(II) complexes [1]; as such, spin transitions can induce dramatic changes in physical properties. SCO(-like) materials are typically developed within coordination chemistry and synthesized via self-assembly methods [2,3,4,5,6], sometimes resulting in magnetic metal–organic frameworks [7,8,9].

The choice of spin sources is critical in research strategy formulation. Compared with systems based on a single type of spin source [1,4,5,6], heterospin systems utilize diverse paramagnetic chromophores and offer nearly limitless combinations of geometries and energy levels, making them particularly attractive for frontier orbital engineering [10,11,12]. These systems show great promise, particularly 3d–2p heterospin compounds, where strong exchange interactions are expected due to direct coordination between the nitroxide (aminoxyl) oxygen atom and a transition metal ion [13]. In metal–radical chemistry, ferromagnetic coupling often arises at direct radical coordination sites [13,14,15,16,17]. Indeed, several transitions between ferro- and antiferromagnetic interactions have been achieved solely through subtle structural perturbations [18,19]. However, magnetically switchable behavior remains relatively rare in nitroxide-based systems.

In copper(II) (3d^9^; *S* = 1/2)–nitroxide heterospin compounds, a few strategies for achieving spin transitions have been explored. Ovcharenko and co-workers have extensively studied copper(II)–nitroxide complexes, known as “breathing crystals” [20,21]. The mechanism involves a Jahn–Teller-driven interchange between axial and equatorial coordination roles [22]. Another mechanism of spin transition has been interpreted as pseudorotation between trigonal bipyramidal and square pyramidal geometries in five-coordinate environments [23].

**Figure 1:**
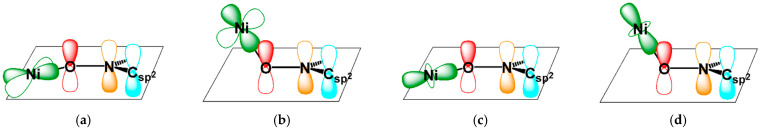
Orbital arrangements illustrating (**a**,**b**) Ni(3d*_x_*^2^_−*y*_^2^)–O(2p_z_)–N(2p_z_)–C_sp_^2^(2p_z_) and (**c**,**d**) Ni(3d*_z_*^2^)–O(2p_z_)–N(2p_z_)–C_sp_^2^(2p_z_) interactions. In (**a**,**c**), the nickel(II) ion is located in-plane within the π* nodal plane of the radical, while in (**b**,**d**), it is displaced out-of-plane.

In contrast, nickel(II) (3d^8^; *S* = 1)–nitroxide heterospin systems present a fundamentally different scenario. High-spin nickel(II) is not a Jahn–Teller ion and typically adopts an octahedral geometry, which precludes pseudorotation. Therefore, changes in total spin (*S*_total_) state may instead arise through internal angular deformation of the ligand framework, without major distortion of the coordination polyhedron. Traditionally, the magnetic behavior of copper(II)– and nickel(II)–nitroxide complexes has been explained by their coordination geometry—weak ferromagnetic coupling in axial coordination (for the copper(II) case) and strong antiferromagnetic coupling in equatorial coordination [13]. However, as reported by Luneau and co-workers [24,25], ferromagnetic coupling is favored when the magnetic orbitals, metal dσ and radical π*, are arranged orthogonally in a planar chelate (Figure 1a,c); in short, Ni(3d*_x_*^2^_−*y*_^2^) or Ni(3d*_z_*^2^) ⊥ O(2p_z_). In contrast, nonplanarity induces orbital overlap, leading to antiferromagnetic coupling (Figure 1b,d); Ni(3d*_x_*^2^_−*y*_^2^) or Ni(3d*_z_*^2^) ∥ O(2p_z_). The dihedral angle between the basal d*_x_*^2^_−*y*_^2^ plane of the coordination polyhedron and the ligand nodal *xy* plane with respect to the π-conjugated O–N–C system indicates the degree of orbital overlap [26,27,28,29]. Although there are a number of geometrical parameters around the Ni, O, N, and C framework, we focus on one specific parameter. Namely, the Ni–O–N–C_2py_ torsion (dihedral) angle serves as a useful structural indicator of the displacement of the metal ion from the nodal plane of the radical π* orbital [30,31]. In this context, a clear correlation between the exchange coupling constant (*J*) and the torsion angle (|*ϕ*|) has been observed in various octahedral copper(II) and nickel(II) complexes [32,33]. Thus, the usefulness of this parameter has been well established.

**Figure 2:**
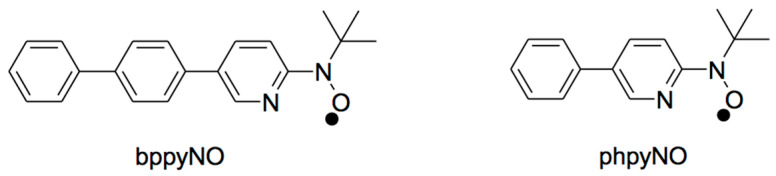
Structural formulas of bppyNO and phpyNO.

In this study, we focus on five-membered nitroxide–nickel(II) chelates, which are sterically predisposed to favor ferromagnetic coupling. The 2p–3d exchange interaction can be tuned through appropriate molecular design strategies, such as expanded π-conjugation. To this end, we introduce a new *S* = 1/2 nitroxide ligand, *tert*-butyl 5-(*p*-biphenylyl)-2-pyridyl nitroxide (abbreviated as bppyNO; see Figure 2), along with its nickel(II) complex, [Ni(bppyNO)_2_Br_2_], as candidates for targeted materials. For structure–property comparison, the corresponding phpyNO and its complexes [34] are also referenced. Complementary theoretical calculations support the observed spin transition behavior and provide deeper insight into the underlying mechanism.

## 2. Materials and Methods

### 2.1. Synthesis of bppyNO

To a tetrahydrofuran (THF) solution (20 mL) of 5-(*p*-biphenylyl)-2-bromopyridine [35] (0.931 g; 3.00 mmol) *n*-BuLi in hexane (2.6 mol/L, Kanto Chemical Co., Inc., Tokyo, Japan) (1.2 mL; 3.1 mmol) was added dropwise by a syringe at −78 °C. After being stirred at −78 °C for 1 h, a THF solution of 2-methyl-2- nitrosopropane (0.400 g; 18.8 mmol) was added through a dropping funnel for 2 h. The reaction mixture was gradually warmed up and stirred overnight. The reaction was quenched by adding an aqueous solution of ammonium chloride. The organic layer was separated, washed, dried over anhydrous MgSO_4_, and filtered. After the filtrate was concentrated under reduced pressure, the precursory hydroxylamine (bppyNOH) precipitated as colorless solids were separated on a filter, washed with a small amount of chilled hexane, and air-dried. The yield was 0.632 g (1.98 mmol; 66%). M.p. 155–156 °C. ^1^H NMR (500 MHz, (CD_2_)_2_SO): δ 1.31 (s, 9H), 7.20 (d, 1H), 7.39 (d, 1H), 7.47 (t, 2H), 7.65 (d, 4H), 7.69 (d, 2H), 7.85 (d, 1H), 8.61 (s, 1H). ^13^C NMR (125 MHz, (CD_3_)_2_SO): 27.9, 61.3, 113.7, 127.0, 127.1, 127.6, 127.8, 128.0, 129.5, 135.6, 137.0, 139.3, 140.1, 144.5, 163.9. FT-IR (neat, attenuated total reflection (ATR)): 3400, 2962, 1467, 1358, 1040, 830, 760 cm^−1^. MALDI-TOF MS: [M + H]^+^ Obs. 319.17225 Calc. 319.18049 for [M + H]^+^.

A dichloromethane solution (1.0 mL) containing the above hydroxylamine (bppyNOH; 31.8 mg, 0.10 mmol) and freshly prepared Ag_2_O (23.1 mg; 0.10 mmol) were mixed, and the resultant suspension was stirred at room temperature for 1 h. After being filtered through celite, the filtrate was concentrated under reduced pressure. The precipitated dark red solids were collected over a filter paper. The yield of bppyNO was 29.8 mg (0.094 mmol; 94%). M.p. 165–166 °C. FT-IR (neat, ATR): 2987, 1076, 828, 762 cm^−1^. MALDI-TOF MS: [M + H]^+^ Obs. 318.17341. Calc. 318.17226 for [M + H] ^+^. ESR (X band, room temperature in toluene): *g* = 2.0067, *a*_N(nitroxide)_ = 0.999 mT, *a*_N(py)_ = 0.136 mT, *a*_H3(py)_ = 0.224 mT, *a*_H4(py)_ = 0.082 mT, *a*_H6(py)_ = 0.082 mT (Figure S4, Supporting Information). The parameters were optimized using the EasySpin software [36]. 

### 2.2. Synthesis of [Ni(bppyNO)_2_Br_2_]

To a dichloromethane solution (1 mL) containing bppyNO (0.0624 g; 0.20 mmol) an acetonitrile solution (2 mL) containing NiBr_2_·3H_2_O (Kanto Chemical Co., Inc.) (27.2 mg; 0.10 mmol) was added through filtration at room temperature. The resultant solution was allowed to stand in a refrigerator (−4 °C) for 3 days. Black precipitates were separated on a filter, washed, and air-dried. The yield of [Ni(bppyNO)_2_Br_2_] was 26.2 mg (30 mmol; 15%). M.p. 223 °C (dec.). FT-IR (neat, ATR) 2987, 1463, 1364, 1076, 828, 762, 545, 461 cm^−1^. Anal. Calcd. for C_42_H_42_Br_2_N_4_NiO_2_: C 59.12, H 4.96, N 6.57%. Found: C 59.54, H 5.06, N 6.62%.

### 2.3. X-Ray Crystallographic Study

Single-crystal X-ray diffraction data for [Ni(bppyNO)_2_Br_2_] were collected on a XtaLAB Synergy R diffractometer equipped with a HyPix detector (Rigaku, Tokyo, Japan) and graphite-monochromated Mo Kα radiation (*λ* = 0.71073 Å). The *hkl* indices and intensity data were extracted using CrysAlisPro [37]. The structure was solved by direct methods and refined using Fourier techniques within Olex2 (version 1.5) [38]. Further refinements were carried out with SHELXL [39]. Thermal displacement parameters for all non-hydrogen atoms were refined anisotropically, while hydrogen atoms were placed at calculated positions and treated as “riding”.

Disorder models were introduced to account for the position of the nitroxide oxygen atom and synchronously dislocated bromine atom at 90 K and the conformation of the outer phenyl ring at 300 K. Occupancy factors were refined to 0.568(13)/0.432(13) and 0.67(3)/0.33(3), respectively. Selected crystallographic data are summarized in Table 1. Full experimental details and geometric parameters are available from CCDC deposition numbers 2 447 889 and 2 447 890 for 90 K and 300 K data, respectively.

### 2.4. Magnetic Study

Magnetic susceptibility measurements for polycrystalline [Ni(bppyNO)_2_Br_2_] were performed using an MPMS3 SQUID magnetometer (Quantum Design Inc., San Diego, CA, USA). Temperature was scanned over the range 1.8–400 K under a static magnetic field of 0.5 T. Diamagnetic corrections were applied using Pascal’s constants [40].

### 2.5. DFT Calculation Study

Density functional theory (DFT) calculations were carried out using Gaussian 16 Revision C.01 [41]. The broken symmetry (BS) approach [42,43] was employed to analyze the energy-level structures of [Ni(bppyNO)_2_Br_2_]. Self-consistent field energies were calculated using experimental coordinates. The unrestricted B3LYP functional was used in single-point calculations with a hybrid basis set: lanl2dz for Ni and Br and 6-311+G(2d,p) for C, H, N, and O [44]. Geometry optimizations were performed with lanl2dz for Ni and Br and 6-31+G(d) for the remaining atoms. The exchange coupling constant *J* in the spin Hamiltonian (Equation (1)) was evaluated by Yamaguchi’s equation (Equation (2)) [45,46].

(1) $$\hat{H}=-2J{\hat{S}}_{1}\cdot {\hat{S}}_{2}$$

(2) $$J=\frac{E_{BS}^{LS}-E^{HS}}{{\;\langle {\hat{S}}^{2}\rangle }^{HS}-{\langle {\hat{S}}^{2}\rangle }_{BS}^{LS}\;}$$

## 3. Results and Discussion

### 3.1. Synthesis and Crystallographic Analysis

A new paramagnetic ligand, bppyNO, was obtained as a crystalline product via oxidation of the corresponding hydroxylamine precursor. The solution-phase X-band ESR spectrum of bppyNO exhibited a characteristic 1/1/1 triplet with a hyperfine coupling constant *a*_N(nitroxide)_ = 0.999 mT at *g* = 2.0067 (measured in toluene at room temperature), typical for aryl *tert*-butyl nitroxide radicals [32]. Further splitting was ascribable to a coupling with the pyridine nitrogen atom, giving *a*_N(py)_ = 0.136 mT. The pyridine hydrogen atoms participate in the hyperfine splitting as well (Figure S4, Supporting Information).

**Figure 3:**
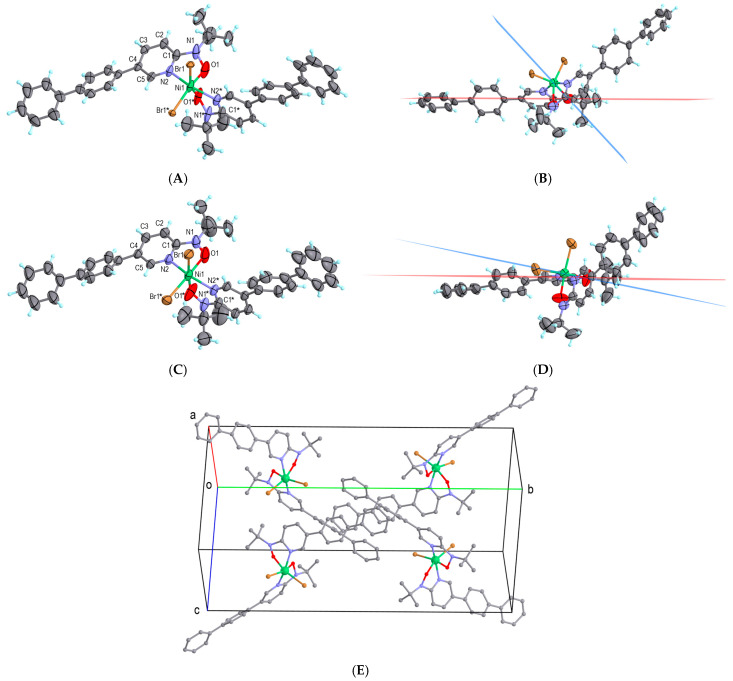
The X-ray crystal structure of [Ni(bppyNO)_2_Br_2_], measured (**A**,**B**) at 90 K and (**C**,**D**) at 300 K. The Ni-O-N and O-N-C_2py_ planes are drawn in blue and red, respectively, depicting *ϕ*(Ni-O-N-C_2py_) in (**B**,**D**). The thermal ellipsoids are drawn at 50% probability. Only the major disorder is shown. Atomic color codes: C, gray; H, turquoise; N, blue; O, red; Ni, green; Br, brown. Symmetry operation code: * (*x*, 1/2—*y*, 1/2—*z*). (**E**) The molecular arrangement in the crystal of [Ni(bppyNO)_2_Br_2_]. Hydrogen atoms are omitted.

The [Ni(bppyNO)_2_Br_2_] complex was synthesized by mixing acetonitrile solutions of bppyNO and nickel(II) bromide. Elemental, spectroscopic, and powder X-ray diffraction analyses (Figure S5, Supporting Information) confirmed the composition and phase purity. Single-crystal X-ray diffraction analysis was performed at 90 and 300 K, and the molecular structures were determined as shown in Figure 3A,B and Figure 3C,D, respectively. A doubly chelated 2p–3d–2p heterospin triad was formed, featuring five-membered chelate rings with direct Ni–nitroxide coordination. The asymmetric unit contains one half of the molecule. The remaining coordination sites are occupied by bromide ligands. To illustrate the dihedral angle (|*ϕ*(Ni-O-N-C_2py_)|), defined between the planes Ni-O-N and O-N-C_2py_, is shown in Figure 3B,D. Selected crystallographic and geometric parameters are summarized in Table 1. The sign of *ϕ* is regulated by stereoisomerism, and the absolute value is important in the discussion on orbital symmetry (Figure 1).

At 90 K, the nitroxide oxygen atom exhibits positional disorder, which disappears at 300 K, although thermal ellipsoids remain relatively large. Significant changes were observed in the dihedral angle *ϕ*. Obviously, the whole molecule hardly moves, but the internal angular torsion is drastically changed. According to a known magneto-structural correlation [32,33], the critical torsion angle *ϕ*_C_ = 21(1)° defines the boundary between regimes favoring ferromagnetic and antiferromagnetic couplings in nickel(II)–radical systems [47]. The observed *ϕ* = 46.9(8)° at 90 K and 10.6(5)° at 300 K indicate ferromagnetic and antiferromagnetic coupling regimes, respectively. A minor disordered component at 90 K with *ϕ* = 26.1(11)° lies close to the critical boundary and thus requires detailed consideration. The torsion angle changes, Δ*ϕ* = 36° and 37°, are among the largest reported for related compounds.

In the crystal structure of [Ni(bppyNO)_2_Br_2_], the molecules are discrete and crystallize in the orthorhombic *Pnna* space group (Figure 3E). No solvent-accessible voids or lattice solvent molecules were detected. The closest intermolecular contacts occur between peripheral phenyl rings. Therefore, the magnetic behavior of this compound is expected to originate primarily from intramolecular interactions.

### 3.2. Magnetic Properties

**Figure 4:**
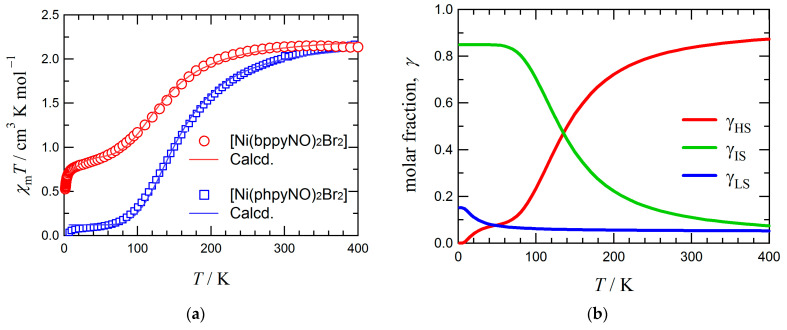
(**a**) The *χ*_m_*T* vs. *T* plots for [Ni(L)_2_Br_2_] (L = bppyNO and phpyNO [34]). The solid lines indicate the best fit by the van’t Hoff equation. For the equation and parameters, see the main text. (**b**) Simulated molar fractions γ_HS_, γ_IS_, and γ_LS_ to reproduce the *χ*_m_*T* vs. *T* profile for [Ni(bppyNO)_2_Br_2_] in (**a**). The γ_HS_ vs. γ_LS_ and γ_HS_ vs. γ_IS_ ratios are regulated by the van’t Hoff law.

Figure 4a shows the temperature dependence of the magnetic susceptibility (*χ*_m_*T*) for [Ni(bppyNO)_2_Br_2_]. At 400 K, the *χ*_m_*T* value was 2.2 cm^3^ K mol^−1^, which is lower than the spin-only value of 3.0 cm^3^ K mol^−1^ expected for a single *S* = 2 species, yet higher than that expected for a combination of two doublet (*S* = 1/2) radicals and one triplet (*S* = 1) ion (1.75 cm^3^ K mol^−1^). This result suggests the presence of intramolecular 2p–3d ferromagnetic coupling. Upon cooling, the *χ*_m_*T* value decreased steadily, indicating the emergence of significant antiferromagnetic interactions. However, a non-zero *χ*_m_*T* value persisted in a low-temperature region, a phenomenon commonly observed in materials with incomplete spin crossover [48,49].

Structural analyses revealed that the coordination geometry of the complex is temperature-dependent. Since the exchange coupling constant *J* varies with molecular conformation, and thus with temperature, the conventional Heisenberg–Dirac–van Vleck model assuming a constant *J* is insufficient for describing the observed magnetic behavior [50,51]. To address this, we applied the van’t Hoff law [52,53] to model the thermally induced structural transformation accompanying spin equilibrium (Equation (3)). The experimental *χ*_m_*T* values were converted to molar high-spin (HS) fractions, *γ*_HS_, using Equation (4), where *C*_0_ represents the Curie constant of the HS state.

(3) $$\gamma_{HS}=\frac{1}{1+exp[\left(\frac{\Delta H}{R}\right)\left(\frac{1}{T}-\frac{1}{T_{1/2}}\right)]}$$

(4) $$\chi_{m}T=C_{0}\gamma_{HS}$$

If *γ*_HS_ approaches 0%, *χ*_m_*T* would also approach zero, as expected for a two-state system (HS, *S_total_* = 2 and LS, *S_total_* = 0). However, the experimental profile could not be reproduced by this binary model alone, since residual paramagnetic properties were clearly evident at low temperatures. This discrepancy necessitated the inclusion of an intermediate-spin (IS, *S_total_* = 1) state. The experimentally observed low-temperature *χ*_m_*T* values were approximately 20% smaller than the theoretical value for a pure triplet species (1.0 cm^3^ K mol^−1^), suggesting a thermally driven population of three spin states (HS, LS, and IS). One possible explanation is as follows: assuming that *g* equals to 2.1 as a typical value of nickel(II)–nitroxide systems [19,34], 85.2% of the molecules participate in an HS/IS equilibrium, while the remaining 14.8% undergo an HS/LS equilibrium. Upon heating, the LS and IS states gradually convert to the HS state, resulting in a full HS population at high temperatures. The crossover temperatures are determined to be *T*_1/2_ = 143(2) K for the HS–IS equilibrium and 48(10) K for the HS–LS equilibrium. Simulations incorporating temperature-dependent molar fractions of each spin state (γ_HS_, γ_LS_, and γ_IS_) successfully reproduced the experimental *χ*_m_*T* vs. *T* profile (Figure 4a), with the corresponding spin state populations shown in Figure 4b. The transition proceeds gradually, indicating that the system undergoes a thermally equilibrated solid-state reaction rather than a discrete phase transition.

The crystal structure analysis at 90 K revealed the presence of multiple conformational isomers in the low-temperature phase (see above). A possible model involves three different conformers. A symmetric conformer with a large torsion angle (46.9(8)°) likely corresponds to the LS *S*_total_ = 0 ground state, with a spin structure of 2p(↓)–3d(↑↑)–2p(↓). Another symmetric conformer with a smaller torsion angle (26.1(11)°) may represent the HS *S*_total_ = 2 ground state: 2p(↑)–3d(↑↑)–2p(↑). A third conformer with unsymmetric torsions on each arm (large on one side, small on the other) could give rise to an IS state, corresponding to 2p(↓)–3d(↑↑)–2p(↑) with *S*_total_ = 1. The observed *χ*_m_*T* plateau, indicating incomplete conversion, appears to result from two successive equilibria. The IS species is suggested to be relatively stable, likely due to steric effects. In the symmetric LS form, the small bond angle ∠O1–Ni1–O1* = 58.4(7)° (Figure S6, Supporting Information) implies significant strain, whereas the IS form relaxes this constraint with a wider angle, ∠O1–Ni1–O1A* = 87.7(7)°, which is sterically more favorable.

Structural analysis confirmed a planar coordination geometry at high temperatures, which transforms into a more distorted geometry upon cooling. This solid-state structural transition involves only modest torsional deformation. In related systems, such as copper(II)–nitroxide–nitroxide–copper(II) and copper(II)–nitroxide compounds, cooperative out-of-plane distortion occurs on every chelate ring [54,55], while in nitroxide–Cu–nitroxide systems, deformation is localized to one side [56]. In nitroxide–nickel(II)–nitroxide systems, where the nickel(II) center has *S* = 1, synchronized deformation occurs on both sides [34,57]. These examples suggest that spin transition is more likely the driving force, rather than a consequence, of the structural changes. An entropy-driven mechanism has previously been proposed [19]. However, the present results appear to contradict this empirical rule, implying an enthalpic disadvantage associated with atomic motions in the bulky biphenyl substituents as well as the interference of the O1/O1* atoms.

To evaluate the effect of ligand structures, we compared these results with those of the closely related complex [Ni(phpyNO)_2_Br_2_], which shows a spin transition at *T*_1/2_ = 134(1) K [34] (Figure 4a). The torsion angle changes are reported to be Δ*ϕ* = 13.8 and 11.3° between 100 and 400 K. The similarity in transition temperatures suggests that the extent of spin delocalization in bppyNO is comparable to that in phpyNO, as confirmed by DFT calculations (see below).

### 3.3. DFT Calculation

**Figure 5:**
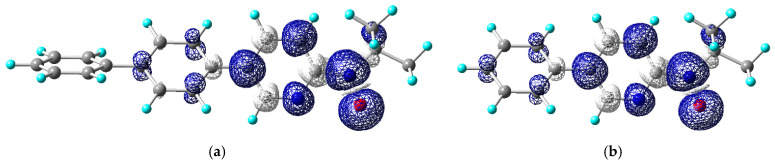
DFT-optimized molecular structures and spin density maps of (**a**) bppyNO and (**b**) phpyNO. Calculated at the UB3LYP/6-311+G(2d,p)//UB3LYP/6-31+G(d) level. Blue and white lobes stand for positive and negative spin densities, respectively. For atomic color codes, see Figure 3.

The molecular design of bppyNO was evaluated using DFT calculations carried out with Gaussian16 [41]. During geometry optimization, the relative configuration of the pyridine nitrogen and nitroxide oxygen atoms was fixed in the *syn* conformation, as found in coordination compounds. The optimized geometries and corresponding spin density distributions, approximately representing the singly occupied molecular orbital, are illustrated in Figure 5.

In the context of developing spin transition materials, it is essential to consider the crossover between ferromagnetic and antiferromagnetic coupling regimes [58,59,60]. For thermally induced spin transitions to occur, a small energy gap between the HS and LS states is desirable. This implies that the exchange coupling must also be small. Since the magnitude of exchange interaction is proportional to the spin densities at the interacting atomic centers [61], bppyNO was initially considered to be more promising than phpyNO. This expectation arose from the presumed enhanced π-conjugation via the additional phenyl substitution in bppyNO.

Contrary to this anticipation, the spin density on the nitroxide oxygen atom in bppyNO was found to be nearly identical to that in phpyNO. The calculated spin densities and isotropic hyperfine coupling constants (*a*_N_) are summarized in Table 2. In bppyNO, the unpaired electron is significantly delocalized onto the inner phenyl ring, whereas the outer phenyl ring contributes minimally to the spin distribution. Consequently, the spin density in the pyridine and inner phenyl moieties is not substantially reduced compared to phpyNO. This is consistent with the comparable *a*_N_ values observed by ESR spectroscopy. The hyperfine constant *a*_N_ is directly proportional to the nitrogen spin density (*ρ*_N_), according to the relationship *a*_N_ = *Q*_N_*ρ*_N_, where *Q*_N_ is a constant [62]. Furthermore, experimental studies using polarized neutron diffraction have confirmed that the oxygen spin density (*ρ*_O_) is approximately proportional to *ρ*_N_ [63]. Taken together, these results indicate that bppyNO offers no significant advantage over phpyNO in terms of spin density distribution for the purposes of this study.

**Figure 6:**
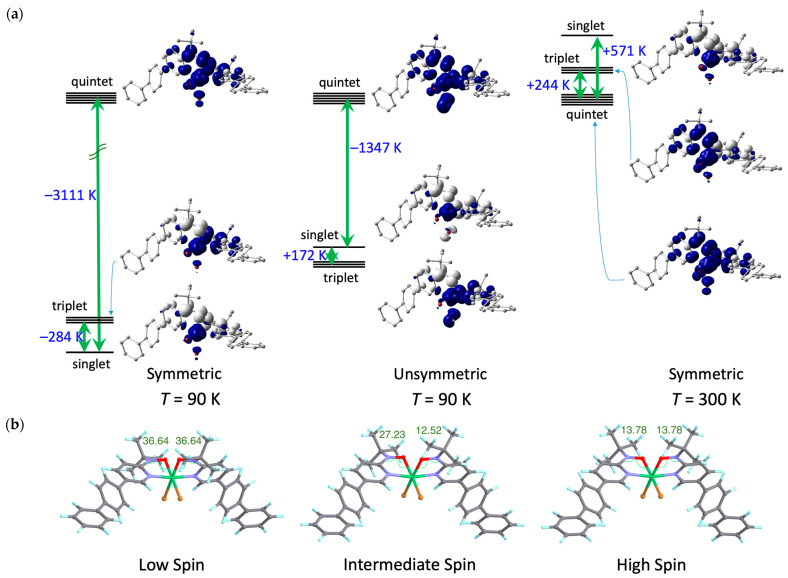
(**a**) Energy level diagrams and spin density maps of the singlet, triplet, and quintet states of [Ni(bppyNO)_2_Br_2_]. (**left**) The symmetrical molecule (*ϕ* = −46.9°) at 90 K, (**center**) the asymmetric molecule (*ϕ* = −46.9 and 26.1°) at 90 K, and (**right**) the symmetric molecule (*ϕ* = −10.6°) at 300 K, calculated at the UB3LYP level with the basis sets lanl2dz for Ni and Br and 6-311+G(2d,p) for the other atoms. Positive (blue) and negative (white) spin densities are drawn with an isocontour of 0.004 e^–^ Å^−3^. (**b**) DFT-optimized structures of [Ni(bppyNO)_2_Br_2_]. (**left**) The *S*_total_ = 0 state, (**center**) *S*_total_ = 1 state, and (**right**) *S*_total_ = 2 state. The optimization was carried out using the UB3LYP method with the basis sets lanl2dz for Ni and Br and 6-31+G(d) for other atoms. The *ϕ* values are written in the figures. For atomic color codes, see Figure 3.

DFT calculations were performed using the experimentally determined atomic coordinates of [Ni(bppyNO)_2_Br_2_] at 90 and 300 K. The calculated energy differences between the singlet and quintet states are illustrated in Figure 6a. At 90 K, the ground state was a singlet, with a singlet–quintet energy gap of −3111 K. The exchange coupling parameter is calculated as 2*J*/*k*_B_ = −1447 K. The triplet state resided between the singlet and quintet levels. At 300 K, the ground state switched to a quintet, with an energy gap of +571 K and 2*J*/*k*_B_ = +282 K, while the triplet state resided between the singlet and quintet levels. Now, we can show calculational evidence of the isomer having unsymmetric torsions on each arm (large on one side, small on the other). As the center of Figure 6a demonstrates, the triplet state is ground. The second lowest level corresponds to the singlet with a relatively small gap at 172 K.

Geometry optimization provided strong support for a structure-dependent exchange interaction switch. The optimized geometries for the various spin states are presented in Figure 6b and Figure S7–S9 (Supporting Information) with the torsion angle *ϕ*(Ni-O-N-C_2py_) = 36.64° for the *S*_total_ = 0 state (left) and 13.78° for the *S*_total_ = 2 state (right). These results clearly indicate that intramolecular 2p–3d antiferromagnetic coupling is favored by large out-of-plane deformation (large *ϕ*), while ferromagnetic coupling is promoted by a more planar coordination geometry (small *ϕ*). A similar computational approach has been used to support the present results, involving related nickel(II) chelate models with two 2-pyridyl nitroxide ligands [64]. The most important finding is that the optimized geometry for the *S*_total_ = 1 state revealed unsymmetric torsions (Figure 6b (center): one chelate ring exhibited a large torsion (*ϕ* = 27.23°), while the other showed a smaller torsion (*ϕ* = 12.52°). Furthermore, as shown in Figure S10 (Supporting Information), the calculated energy diagram qualitatively reproduces the experimental energy profile presented in Figure 6a. Thus, the proposed intermediate-spin (IS) mechanism (see above) is totally supported by the DFT calculations.

The torsion angles obtained from DFT calculations are somewhat smaller than those observed experimentally. This discrepancy likely arises because the calculations were performed on isolated molecules in the gas phase, neglecting intermolecular interactions and crystal packing effects. For example, in the rigid crystal lattice, the reorientation of the long axis of the linear triaryl group is not easily accommodated. Despite this limitation, the DFT results support a plausible mechanism involving the IS state that accounts for the incomplete spin transition-like behavior observed in this nitroxide–nickel(II)–nitroxide heterospin triad system. A triplet intermediate state is proposed here for the first time among nitroxide–nickel(II)–nitroxide compounds.

## 4. Concluding Remarks

A novel ligand bppyNO and its complex [Ni(bppyNO)_2_Br_2_] were synthesized. A relatively planar chelate geometry at 300 K was characterized, while significant out-of-plane distortion was found at 90 K. The magnetic measurements indicate gradual and incomplete spin transition-like behavior. A three-state model involving an IS triplet state is proposed to explain the *χ*_m_*T* plateau in a low-temperature region. The DFT calculations using the determined structures support the proposed logic. Furthermore, geometry optimizations assuming *S*_total_ = 0, 1, and 2 are in good agreement with the model.

Although numerous studies have been reported on metal–radical heterospin materials, most are based on the assumption of static structures. A single-temperature X-ray diffraction analysis may overlook structural transitions. In contrast, a combined investigation involving structural chemistry, magnetic measurements, and DFT calculations enables a more comprehensive understanding. Our findings demonstrate the existence of spin state switching and support a crossover mechanism between ferro- and antiferromagnetic coupling.

## Figures and Tables

**Table 1:** Selected crystallographic parameters of [Ni(bppyNO)Br_2_].

*T*/K	90	300
Formula, formula weight	C_42_H_42_Br_2_N_4_NiO_2_, 853.32	
Crystal system	orthorhombic	
Space group	*Pnna*	
*a/*Å	9.8907(6)	10.1957(6)
*b/*Å	28.9625(13)	29.2241(12)
*c/*Å	14.3574(7)	14.3493(6)
*V/*Å^3^	4112.8(4)	4275.5(4)
*Z*	4	4
*d*_calcd_/g·cm^−3^	1.378	1.326
*μ* (MoKα)/mm^−1^	2.453	2.360
No. of unique reflections	23 447	24 950
*R*(*F*) (*I* > 2σ(*I*)) ^a^	0.0696	0.0484
*wR*(*F*^2^) (all reflections) ^b^	0.1608	0.1325
Goodness-of-fit parameter	1.079	0.995
CCDC reference	2 447 889	2 447 890
geometry		
*d*(Ni1-O1)/Å	2.003(8), 2.131(8) ^c^	2.032(3)
*d*(Ni1-N2)/Å	2.058(4)	2.061(2)
*d*(O1-N1)/Å	1.392(8), 1.344(8) ^c^	1.274(4)
*d*(N1-C1_2py_)/Å	1.399(7)	1.408(4)
*θ*(Ni1-O1-N1)/deg	111.9(5), 107.1(5) ^c^	117.0(2)
*θ*(O1-Ni1-N2)/deg	74.4(3), 78.6(2) ^c^	76.51(10
*ϕ*(Ni1-O1-N1-C1_2py_) /deg	−46.9(8), 26.1(11) ^c^	−10.6(5)

^a^ *R* = Σ[|*F*_o_| − |*F*_c_|]/Σ|*F*_o_|. ^b^ *wR* = [Σ*w*(*F*_o_^2^ − *F*_c_^2^)/Σ*wF*_o_^4^]^1/2^. ^c^ Geometries related to the major and minor O1 disordered positions.

**Table 2:** Experimental hyperfine coupling constants (*a*_N_) and DFT calculated spin densities (*ρ* ^a^).

Compound	*a*_N_/mT	*ρ*_N_	*ρ*_O_
bppyNO	0.999	0.3523	0.5284
phpyNO	1.000 ^b^	0.3519	0.5297

^a^ For calculation details, see Figure 5. ^b^ Ref. [32].

## Data Availability

The original contributions presented in this study are included in the article/supplementary material. Further inquiries can be directed to the corresponding author.

## Supplementary Materials

The following supporting information can be downloaded at: https://www.mdpi.com/article/10.3390/ma18122793/s1, Figure S1: ^1^H and ^13^C NMR spectra of bppyNOH; Figure S2: FT-IR spectra of bppyNOH, bppyNO, and [Ni(bppyNO)_2_Br_2_]; Figure S3: HRMS spectra of bppyNOH and bppyNO; Figure S4: ESR spectrum of bppyNO; Figure S5: PXRD of [Ni(bppyNO)_2_Br_2_]; Figure S6: Disorder in [Ni(bppyNO)_2_Br_2_]. Figures S7–S9: DFT-optimized structures and Cartesian coordinates of LS, IS, and HS [Ni(bppyNO)_2_Br_2_], respectively; Figure S10: Energy level diagrams of the DFT-optimized structures of [Ni(bppyNO)_2_Br_2_].

## Abbreviations

The following abbreviations are used in this manuscript:

bppyNO	*tert*-butyl 5-(*p*-biphenylyl)-2-pyridyl nitroxide
phpyNO	*tert*-butyl 5-phenyl-2-pyridyl nitroxide
DFT	Density functional theory
SCO	Spin crossover
HS	High spin
IS	Intermediate spin
LS	Low spin
