# Supplementary material for: Solid-State Transformation (Stotal = 0, 1, and 2) in a Ni2+ Chelate with Two tert-Butyl 5-(p-Biphenylyl)-2-pyridyl Nitroxides
Source: Materials (Basel). 2025 Jun 13;18(12):2793. doi: 10.3390/ma18122793 (PMC12195258; doi:10.3390/ma18122793)
Supplement: Supplementary file 1 [file materials-18-02793-s001.zip › materials-3660849-supplementary.pdf]

# Supporting Information

# Solid–State Transformation ($S_{total}$ = 0, 1, and 2) in a $Ni^{2+}$ Chelate with Two *tert*-Butyl 5-(*p*-Biphenylyl)-2-pyridyl Nitroxides

Masataka Mitsui and Takayuki Ishida *

*The University of Electro-Communications, 1-5-1 Chofugaoka, Chofu, Tokyo 182-8585, Japan*

*E-mail: takayuki.ishida@uec.ac.jp*

Table of Contents

| | | |
|---|---|---|
| **Figure S1.** | $^{1}$H and $^{13}$C NMR spectra of bppyNOH | p. S2 |
| **Figure S2.** | FT-IR spectra of bppyNOH, bppyNO, and [Ni(bppyNO)$_2$Br$_2$] | p. S3 |
| **Figure S3.** | HRMS spectra of bppyNOH and bppyNO | p. S4 |
| **Figure S4.** | ESR spectrum of bppyNO | p. S5 |
| **Figure S5.** | PXRD profiles of [Ni(bppyNO)$_2$Br$_2$] | p. S6 |
| **Figure S6.** | Disorder of X-ray crystal structure of [Ni(bppyNO)$_2$Br$_2$] | p. S7 |
| **Figure S7.** | DFT optimized structure and Cartesian coordinates of LS [Ni(bppyNO)$_2$Br$_2$] | p. S8,9 |
| **Figure S8.** | DFT optimized structure and Cartesian coordinates of IS [Ni(bppyNO)$_2$Br$_2$] | p. S10,11 |
| **Figure S9.** | DFT optimized structure and Cartesian coordinates of HS [Ni(bppyNO)$_2$Br$_2$] | p. S12,13 |
| **Figure S10.** | Energy level diagrams on the DFT optimized structures of [Ni(bppyNO)$_2$Br$_2$] | p. S14 |
| | | |
| **References** | | p. S15 |

(a)

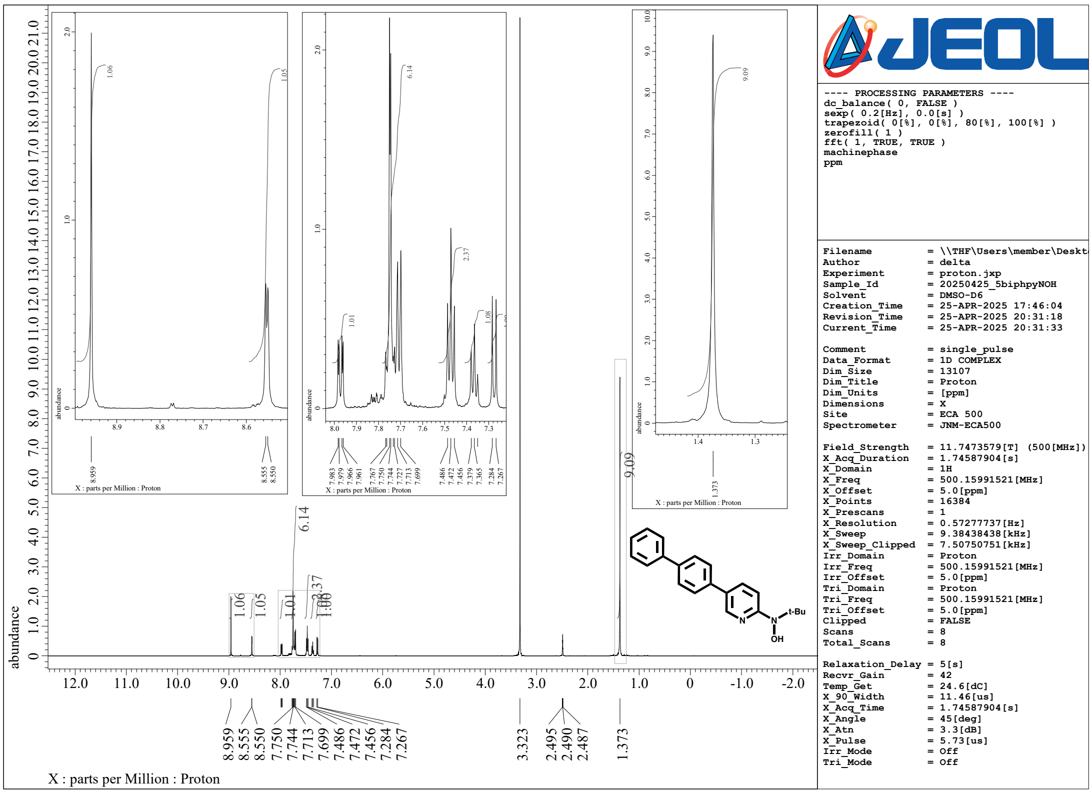


(b)

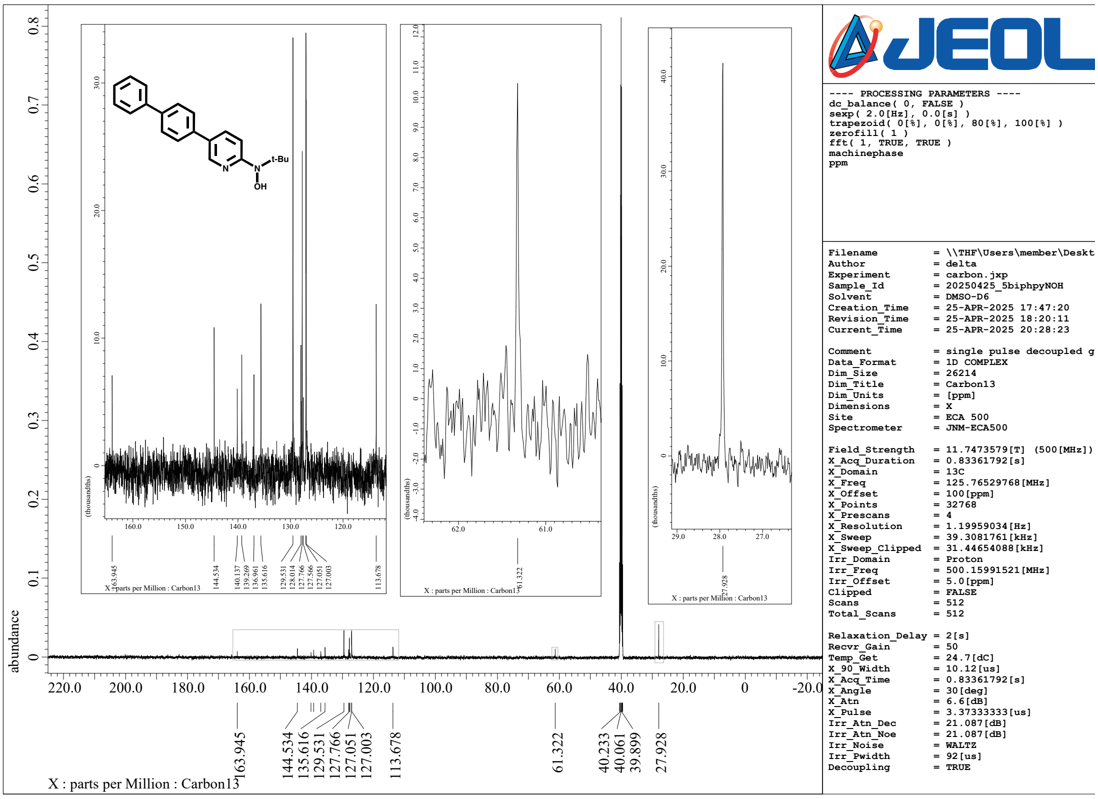


**Figure S1.** (a) $^{1}$H and (b) $^{13}$C{$^{1}$H} NMR spectra bppyNOH, measured in $(CD_2)_2SO$ at 500 MHz for $^{1}$H and 125 MHz for $^{13}$C NMR at room temperature, using a 500 MHz superconducting Fourier transform NMR (ECA-500, JEOL Ltd.). The analysis was performed using Delta Software (JEOL Ltd.).

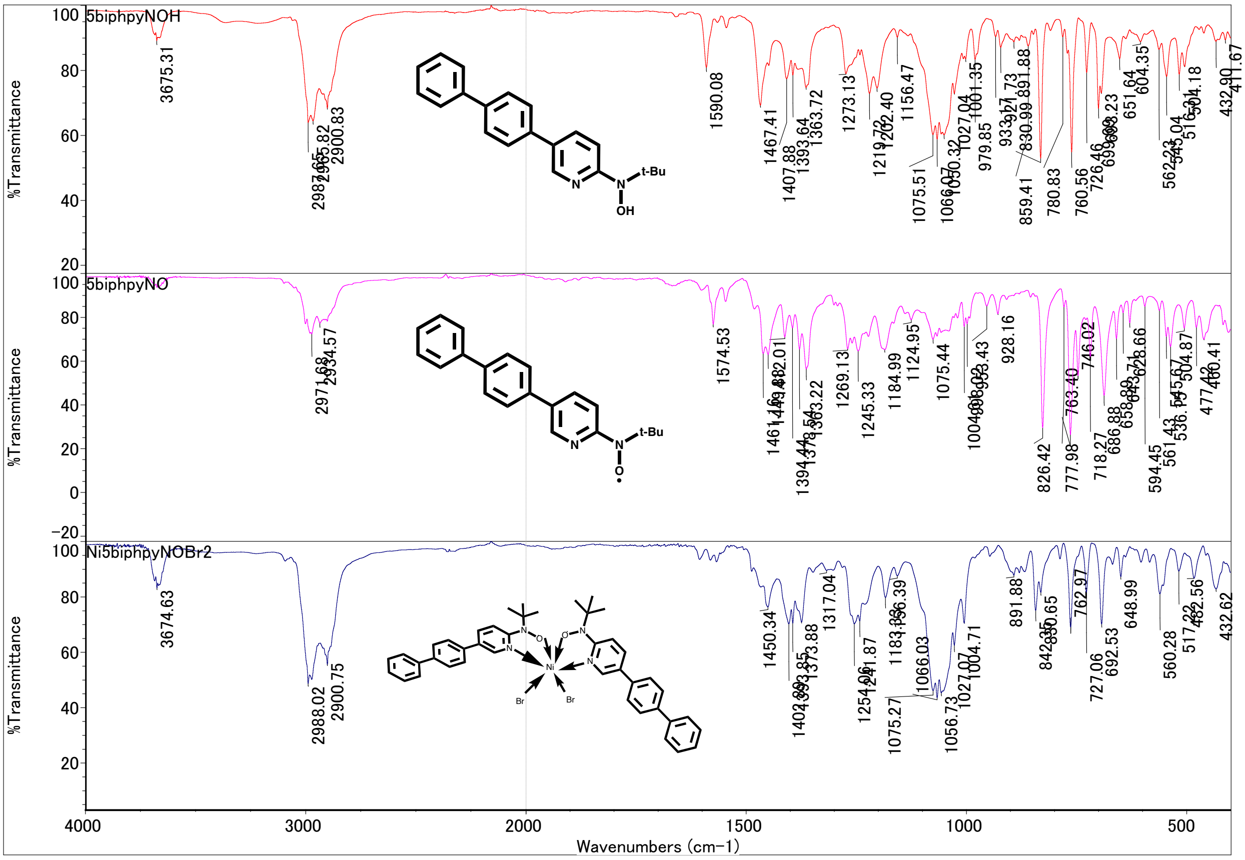


**Figure S2.** FT-IR spectra (neat, ATR) of bppyNOH, bppyNO, and [Ni(bppyNO)$_2$Br$_2$]. Powder samples were placed on a single reflection horizontal ATR accessory, and the spectra were recorded on a Nicolet 6700 spectrometer (ThermoScientific).

(a)

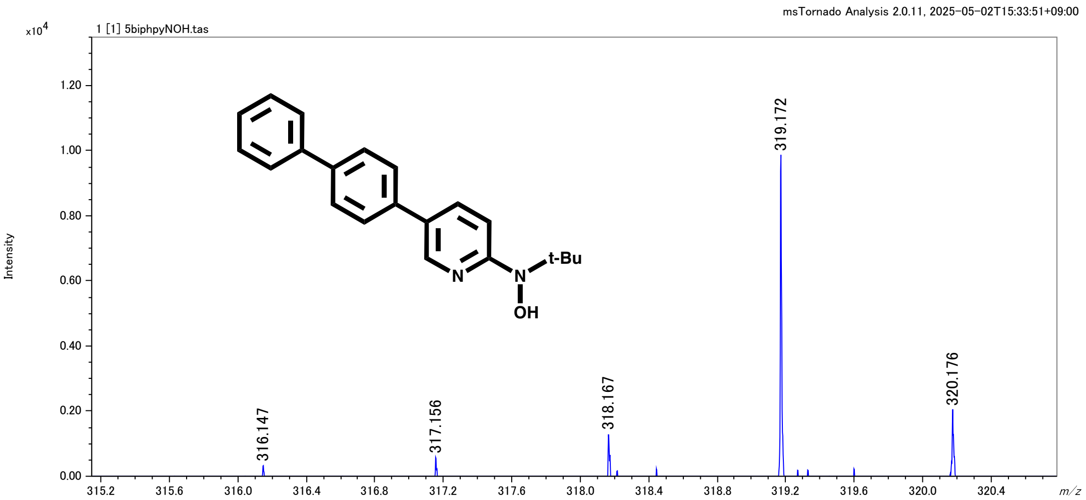


Elemental Composition Estimation

Parameters:

| Mass | Tolerance | Electron Mode | Charge | DBE Range | Max Results |
|---|---|---|---|---|---|
| 319.17225 | 10.00 mDa | Odd/Even | +1 | -0.5 – 200.0 | 100 |

Elements

C 20 – 24 H 20 – 24 N 2 – 2 O 1 – 1 Na 0 – 0

Results:

| # | Formula | Mass | DBE | Abs. Error (mDa) | Error (mDa) | Error (ppm) |
|---|---|---|---|---|---|---|
| 1 | C21 H23 N2 O | 319.18049 | 11.5 | 8.2 | -8.2 | -25.82 |

(b)

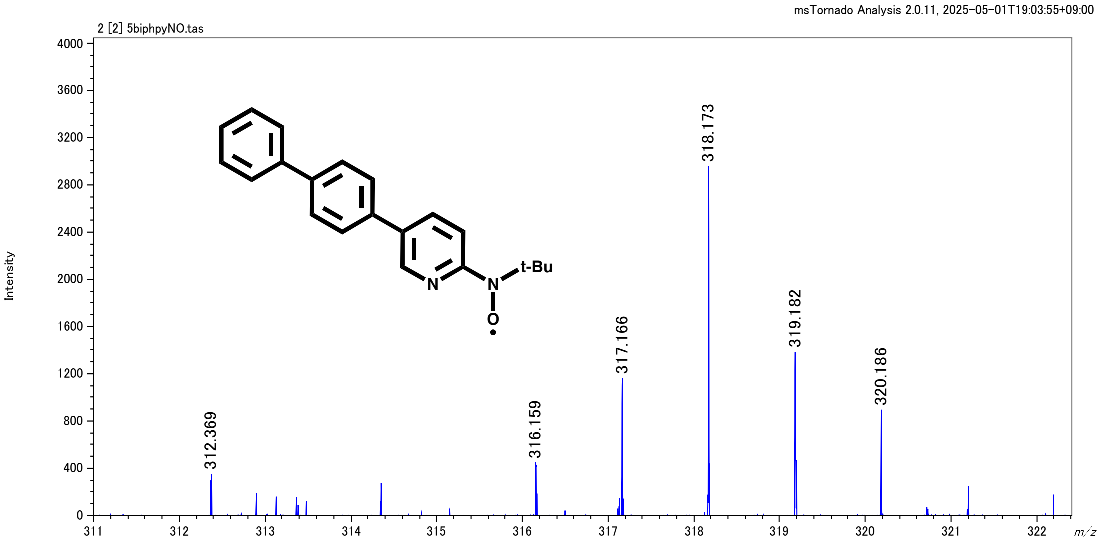


Elemental Composition Estimation

Parameters:

| Mass | Tolerance | Electron Mode | Charge | DBE Range | Max Results |
|---|---|---|---|---|---|
| 318.17341 | 5.00 mDa | Odd/Even | +1 | -0.5 – 200.0 | 100 |

Elements

C 21 – 21 H 22 – 22 N 0 – 10 O 0 – 10 Na 0 – 0

Results:

| # | Formula | Mass | DBE | Abs. Error (mDa) | Error (mDa) | Error (ppm) |
|---|---|---|---|---|---|---|
| 1 | C21 H22 N2 O | 318.17266 | 12.0 | 0.7 | 0.7 | 2.35 |

**Figure S3.** HRMS of (a) bppyNOH and (b) bppyNO measured in MALDI-TOF mode, recorded on a JMS-S3000 SpiralTOF (JEOL Ltd.). Silver trifluoroacetate was used as a cationizing agent, and α-cyano-4-hydroxycinnamic acid was used as a matrix and a reference.

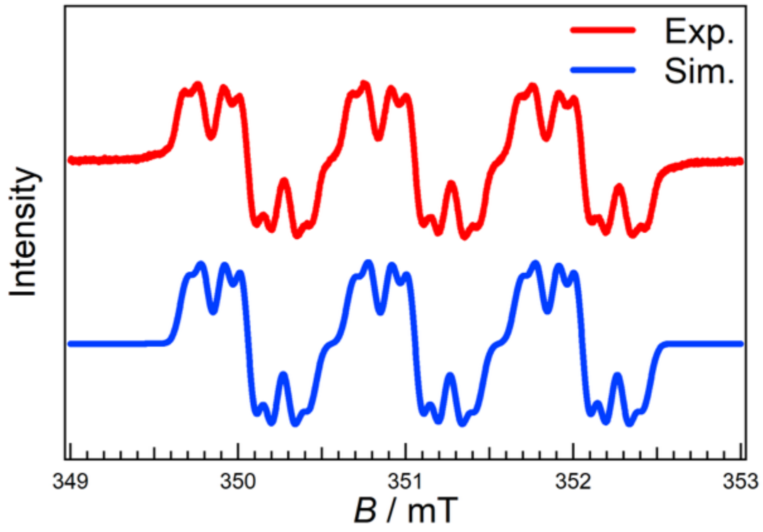


**Figure S4.** ESR experimental spectrum of bppyNO in toluene solution (9.85 GHz, microwave output 0.63 mW, modulation amplitude 0.06 mT) and simulated spectrum. The spectrum was recorded on a Bruker ELEXYS X-band spectrometer. The toluene solution sample was placed in a quartz tube and degassed with a nitrogen stream. Simulation work involving hyperfine splitting constants was performed on the EasySpin program package [S1] running on the MatLab platform. The parameters are as follows: $g$ = 2.0067, $a_{\mathrm{N(nitroxide)}}$ = 0.999 mT, $a_{\mathrm{N(py)}}$ = 0.136 mT, $a_{\mathrm{H3(py)}}$ = 0.224 mT, $a_{\mathrm{H4(py)}}$ = 0.082 mT, $a_{\mathrm{H6(py)}}$ = 0.082 mT.

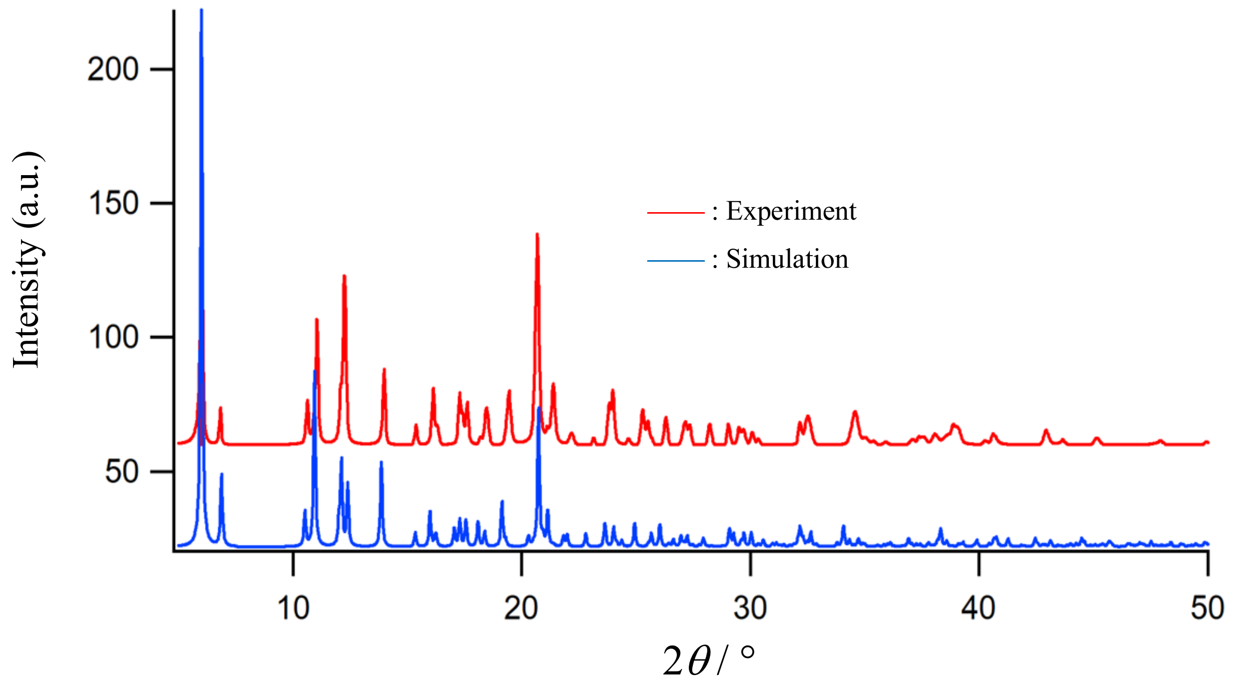


**Figure S5.** PXRD for [Ni(bppyNO)$_2$Br$_2$], recorded on an Ultima III diffractometer (Rigaku) using Cu Kα radiation ($\lambda$ = 1.5418 Å) at room temperature. Simulation data from the single-crystal XRD results are also shown.

(a) (b)

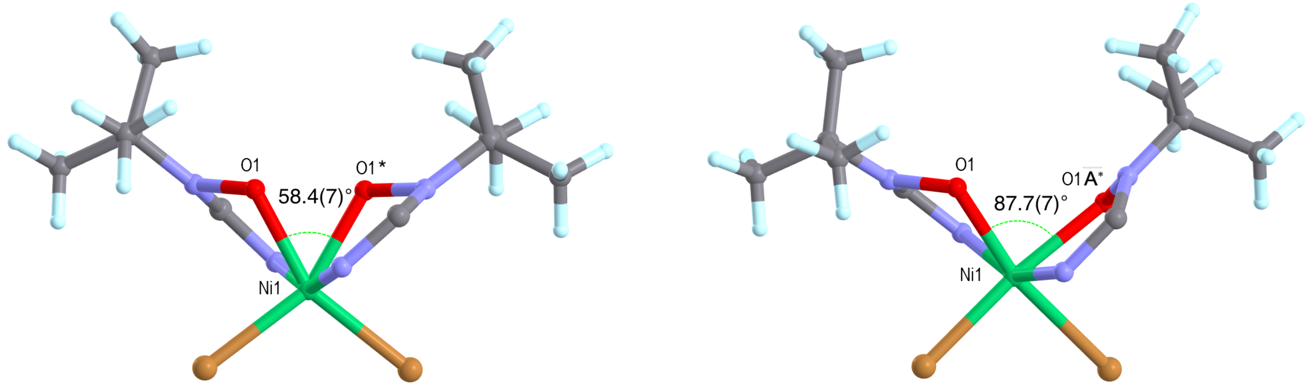


**Figure S6.** Detailed disorder of the X-ray crystal structure of [Ni(bppyNO)$_2$Br$_2$]. (a) Symmetrical position having O1 and O1*. (b) Unsymmetrical position having O1 and O1A*. The O1-Ni1-O1(A)* angles are noted. Biphenyl groups are omitted for clarity. Atomic color codes: C, gray; H, turquoise; N, blue; O, red; Ni, green; Br, brown.

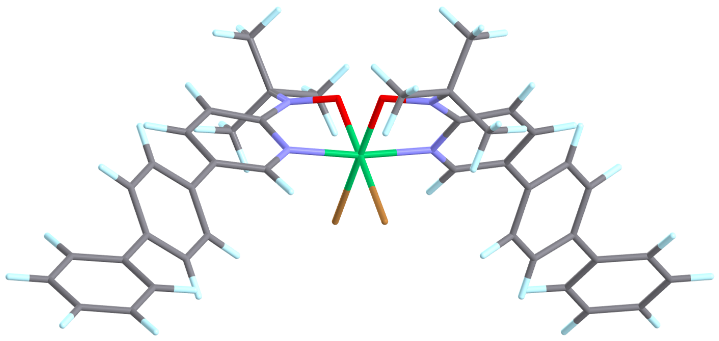

**Figure S7.** DFT optimized structure of LS singlet [Ni(bppyNO)$_2$Br$_2$]. For the calculation protocol and atomic color codes, see Figure S6. The Cartesian coordinates are shown below.

| | | | |
|---|---|---|---|
| Br | -5.85708646 | -8.96093005 | -2.92746149 |
| Ni | -7.60436877 | -7.24729992 | -3.58094399 |
| O | -9.11397298 | -6.10530483 | -4.16767076 |
| N | -9.01910071 | -5.83504379 | -5.46219995 |
| N | -7.74906552 | -7.75006646 | -5.48221322 |
| C | -8.53315512 | -6.88761190 | -6.19675115 |
| C | -8.78607957 | -7.13660053 | -7.55830527 |
| H | -9.45019929 | -6.49026445 | -8.11639089 |
| C | -8.21411342 | -8.24374184 | -8.16119278 |
| H | -8.39537081 | -8.42326686 | -9.21721959 |
| C | -7.40570505 | -9.13150743 | -7.42476612 |
| C | -7.23458754 | -8.83633622 | -6.06671331 |
| H | -6.64500392 | -9.46905758 | -5.41303912 |
| C | -9.28767478 | -4.42838622 | -5.93101215 |
| C | -9.18982546 | -3.50419903 | -4.70676269 |
| H | -8.19687431 | -3.56483362 | -4.25488679 |
| H | -9.36684419 | -2.47669402 | -5.04328407 |
| H | -9.93445754 | -3.75679677 | -3.94843934 |
| C | -10.72276966 | -4.37394946 | -6.49928353 |
| H | -11.44475994 | -4.71379005 | -5.74926406 |
| H | -10.96969197 | -3.34207897 | -6.77450275 |
| H | -10.84137665 | -4.99335160 | -7.39465377 |
| C | -8.24738777 | -3.98942818 | -6.97738094 |
| H | -8.30361084 | -4.56399468 | -7.90594625 |
| H | -8.42836469 | -2.93940451 | -7.23271780 |
| H | -7.23644501 | -4.08191613 | -6.57032891 |
| C | -6.75371158 | -10.31209361 | -8.03329689 |
| C | -7.37851862 | -11.03852189 | -9.06302194 |
| H | -8.37435242 | -10.75721466 | -9.39643674 |
| C | -6.76370219 | -12.14870962 | -9.63667622 |
| H | -7.29086814 | -12.70779957 | -10.40521383 |
| C | -5.49702140 | -12.58360685 | -9.20781399 |
| C | -4.87466801 | -11.85644651 | -8.17760797 |
| H | -3.88117820 | -12.14255010 | -7.84322938 |
| C | -5.48646871 | -10.74537183 | -7.60250104 |
| H | -4.95730406 | -10.19027775 | -6.83274257 |
| C | -4.84195380 | -13.76793644 | -9.81952564 |
| C | -4.93675664 | -14.01434354 | -11.20122739 |
| H | -5.47432028 | -13.31328783 | -11.83452037 |
| C | -4.32192734 | -15.12787678 | -11.77616122 |
| H | -4.40120964 | -15.29234030 | -12.84798264 |
| C | -3.59724690 | -16.02079232 | -10.98155639 |
| H | -3.11772323 | -16.88767387 | -11.42879006 |

| | | | |
|---|---|---|---|
| C | -3.49380594 | -15.78893415 | -9.60698144 |
| H | -2.93976525 | -16.48078345 | -8.97738043 |
| C | -4.10942932 | -14.67542637 | -9.03293927 |
| H | -4.04255925 | -14.52081263 | -7.95921451 |
| Br | -5.85706175 | -5.53369047 | -4.23441103 |
| O | -9.11399013 | -8.38927686 | -2.99422580 |
| N | -9.01911097 | -8.65954980 | -1.69970024 |
| N | -7.74907060 | -6.74453295 | -1.67967621 |
| C | -8.53316793 | -7.60698434 | -0.96514316 |
| C | -8.78611603 | -7.35798231 | 0.39640459 |
| H | -9.45025851 | -8.00430284 | 0.95448108 |
| C | -8.21415004 | -6.25084238 | 0.99929461 |
| H | -8.39542484 | -6.07130821 | 2.05531687 |
| C | -7.40572082 | -5.36308886 | 0.26287615 |
| C | -7.23458961 | -5.65826599 | -1.09517351 |
| H | -6.64499593 | -5.02554976 | -1.74884362 |
| C | -9.28767536 | -10.06621855 | -1.23091547 |
| C | -9.19005454 | -10.99034375 | -2.45522993 |
| H | -8.19717091 | -10.92972591 | -2.90725663 |
| H | -9.36706571 | -12.01785986 | -2.11873816 |
| H | -9.93479336 | -10.73767145 | -3.21342348 |
| C | -10.72268626 | -10.12061469 | -0.66242892 |
| H | -11.44477344 | -9.78069858 | -1.41232082 |
| H | -10.96961857 | -11.15248699 | -0.38722530 |
| H | -10.84112365 | -9.50125077 | 0.23299072 |
| C | -8.24724732 | -10.50529268 | -0.18473486 |
| H | -8.30330698 | -9.93080214 | 0.74388613 |
| H | -8.42822446 | -11.55533010 | 0.07054490 |
| H | -7.23636193 | -10.41281266 | -0.59193179 |
| C | -6.75372762 | -4.18250428 | 0.87141025 |
| C | -7.37854450 | -3.45606619 | 1.90112240 |
| H | -8.37438507 | -3.73736479 | 2.23452422 |
| C | -6.76372875 | -2.34587955 | 2.47477963 |
| H | -7.29090206 | -1.78678181 | 3.24330653 |
| C | -5.49703881 | -1.91099321 | 2.04593330 |
| C | -4.87467547 | -2.63816346 | 1.01574028 |
| H | -3.88117888 | -2.35206847 | 0.68137454 |
| C | -5.48647543 | -3.74923712 | 0.44063055 |
| H | -4.95730334 | -4.30433896 | -0.32911718 |
| C | -4.84197219 | -0.72666455 | 2.65764772 |
| C | -4.93679324 | -0.48024902 | 4.03934670 |
| H | -5.47437055 | -1.18129760 | 4.67263594 |
| C | -4.32196494 | 0.63328351 | 4.61428307 |
| H | -4.40126154 | 0.79775361 | 5.68610241 |
| C | -3.59726728 | 1.52618981 | 3.81968359 |
| H | -3.11774438 | 2.39307077 | 4.26691923 |
| C | -3.49380812 | 1.29432320 | 2.44511145 |
| H | -2.93975391 | 1.98616540 | 1.81551448 |
| C | -4.10943051 | 0.18081622 | 1.87106673 |
| H | -4.04254610 | 0.02619599 | 0.79734379 |

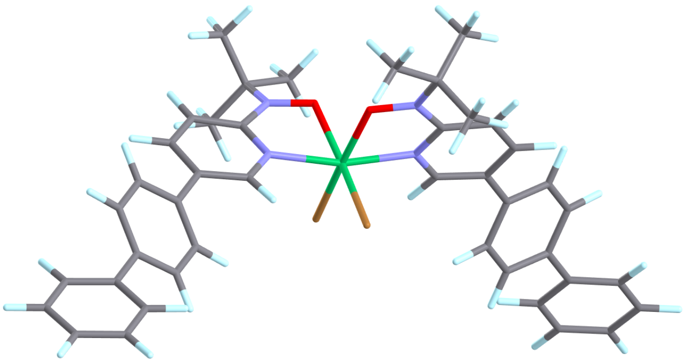

**Figure S8.** DFT optimized structure of IS triplet [Ni(bppyNO)$_2$Br$_2$]. For the calculation protocol and atomic color codes, see Figure S6. The Cartesian coordinates are shown below.

| | | | |
|---|---|---|---|
| Br | -5.91374764 | -9.03791084 | -2.90552198 |
| Ni | -7.61506234 | -7.20373339 | -3.59426102 |
| N | -9.27250835 | -5.96459808 | -5.63899907 |
| N | -7.86011231 | -7.80797597 | -5.60654400 |
| C | -8.65312045 | -7.01328271 | -6.35202202 |
| C | -8.84706471 | -7.26386909 | -7.71827672 |
| H | -9.50619424 | -6.65245669 | -8.31703876 |
| C | -8.17483095 | -8.33038811 | -8.30209374 |
| H | -8.29970202 | -8.51278640 | -9.36564428 |
| C | -7.32839342 | -9.15082351 | -7.53872645 |
| C | -7.22526429 | -8.83419147 | -6.17298279 |
| H | -6.61427761 | -9.41671634 | -5.48936997 |
| C | -9.77737641 | -4.65633917 | -6.22780423 |
| C | -9.92746166 | -3.66208656 | -5.06481471 |
| H | -8.97422478 | -3.51733503 | -4.55034146 |
| H | -10.25630219 | -2.70456528 | -5.48309547 |
| H | -10.66919269 | -3.99618736 | -4.33554677 |
| C | -11.15661034 | -4.89050379 | -6.87613293 |
| H | -11.85747793 | -5.31010361 | -6.14627071 |
| H | -11.55675412 | -3.93016000 | -7.21988711 |
| H | -11.12121240 | -5.55999138 | -7.74008829 |
| C | -8.73838821 | -4.09637329 | -7.21915425 |
| H | -8.64320600 | -4.68765037 | -8.13267098 |
| H | -9.04882576 | -3.08755007 | -7.51238888 |
| H | -7.75636780 | -4.03399798 | -6.73978480 |
| C | -6.57746958 | -10.28654663 | -8.11572280 |
| C | -7.09856957 | -11.03944732 | -9.18338659 |
| H | -8.08927199 | -10.81602667 | -9.57135358 |
| C | -6.38645998 | -12.10529227 | -9.72718781 |
| H | -6.83407064 | -12.68877057 | -10.52731847 |
| C | -5.12158206 | -12.46573876 | -9.22890837 |
| C | -4.60384111 | -11.71267916 | -8.16004304 |
| H | -3.61641261 | -11.94267758 | -7.76959426 |
| C | -5.31382828 | -10.64727861 | -7.61273978 |
| H | -4.86593713 | -10.07296762 | -6.80669376 |
| C | -4.36211403 | -13.60271915 | -9.80880552 |
| C | -4.36322777 | -13.84152086 | -11.19506353 |
| H | -4.90558600 | -13.16844196 | -11.85409892 |
| C | -3.64950124 | -14.91013415 | -11.74012922 |
| H | -3.65702311 | -15.06907630 | -12.81566981 |
| C | -2.91826133 | -15.76470969 | -10.91014947 |
| H | -2.36177432 | -16.59659982 | -11.33409295 |
| C | -2.90768350 | -15.53990090 | -9.53056248 |

| | | | |
|---|---|---|---|
| H | -2.34903283 | -16.20249009 | -8.87419949 |
| C | -3.62155185 | -14.47099382 | -8.98635517 |
| H | -3.62506866 | -14.32215748 | -7.90979062 |
| Br | -6.08560070 | -5.25825248 | -4.30539987 |
| O | -9.08149250 | -8.55360507 | -2.83013840 |
| N | -9.21007755 | -8.56495391 | -1.54574816 |
| N | -7.87127207 | -6.66562913 | -1.57802370 |
| C | -8.59059012 | -7.52498187 | -0.82657428 |
| C | -8.70163383 | -7.34039516 | 0.56220812 |
| H | -9.27223307 | -8.01461444 | 1.18162042 |
| C | -8.04111162 | -6.27079549 | 1.15073791 |
| H | -8.09948400 | -6.14654001 | 2.22831145 |
| C | -7.28304526 | -5.37743158 | 0.37618469 |
| C | -7.24649398 | -5.63729253 | -1.00383564 |
| H | -6.69869821 | -5.00315313 | -1.69458030 |
| C | -9.88598136 | -9.79211373 | -0.95511435 |
| C | -10.24046376 | -10.72963801 | -2.12140992 |
| H | -9.34788150 | -11.04392014 | -2.66670598 |
| H | -10.72748738 | -11.61566599 | -1.69997622 |
| H | -10.92761396 | -10.25816421 | -2.82833932 |
| C | -11.19136046 | -9.38095386 | -0.24478248 |
| H | -11.85811347 | -8.86247853 | -0.94242872 |
| H | -11.70270083 | -10.28433033 | 0.10547936 |
| H | -11.03870875 | -8.73417432 | 0.62185451 |
| C | -8.89132536 | -10.52356115 | -0.02980460 |
| H | -8.66454740 | -9.97835036 | 0.88884786 |
| H | -9.32441312 | -11.48798732 | 0.25752878 |
| H | -7.95115254 | -10.70721518 | -0.55963017 |
| C | -6.55332121 | -4.23039825 | 0.95650900 |
| C | -7.04539022 | -3.54807075 | 2.08383675 |
| H | -7.99914342 | -3.83711447 | 2.51831266 |
| C | -6.35454959 | -2.46988344 | 2.63064767 |
| H | -6.78121181 | -1.94225436 | 3.47951643 |
| C | -5.14091095 | -2.02579641 | 2.07566428 |
| C | -4.65191732 | -2.70876991 | 0.94771448 |
| H | -3.70163465 | -2.41378061 | 0.51145691 |
| C | -5.34063270 | -3.78648211 | 0.39740290 |
| H | -4.91369281 | -4.30580734 | -0.45592502 |
| C | -4.40475117 | -0.87491480 | 2.65813332 |
| C | -4.33565030 | -0.69021235 | 4.05093339 |
| H | -4.80446290 | -1.41522063 | 4.71129781 |
| C | -3.64425916 | 0.39178811 | 4.59835324 |
| H | -3.59608049 | 0.50844349 | 5.67825260 |
| C | -3.00603894 | 1.31423165 | 3.76429265 |
| H | -2.46696881 | 2.15660851 | 4.19003428 |
| C | -3.06584720 | 1.14367632 | 2.37820377 |
| H | -2.58001112 | 1.85891032 | 1.71915790 |
| C | -3.75736193 | 0.06135907 | 1.83165066 |
| H | -3.81709045 | -0.04575855 | 0.75178285 |
| O | -9.26680800 | -6.06212804 | -4.35227652 |

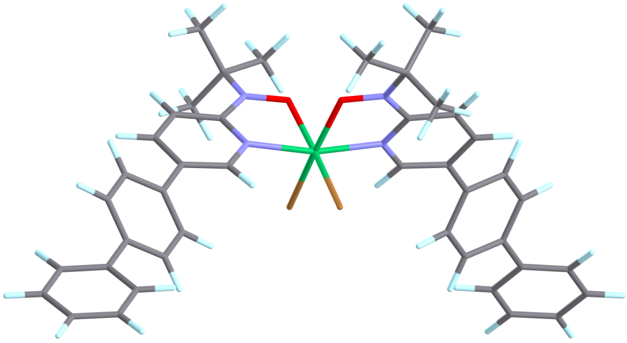

**Figure S9.** DFT optimized structure of HS quintet [Ni(bppyNO)$_2$Br$_2$], calculated using the UB3LYP method with a hybrid basis set: lanl2dz for Ni and Br and 6-31+G(d) for C, H, N, and O [S2,S3]. The number of imaginary frequencies was zero. Atomic color codes: C, gray; H, turquoise; N, blue; O, red; Ni, green; Br, brown. The Cartesian coordinates are shown below.

| | | | |
|---|---|---|---|
| Br | -6.19793561 | -9.24720626 | -2.82562024 |
| Ni | -7.82802009 | -7.36397662 | -3.56888620 |
| O | -9.35249666 | -6.09989388 | -4.35185262 |
| N | -9.47268816 | -6.11310321 | -5.63784647 |
| N | -8.06809511 | -7.96211889 | -5.56757015 |
| C | -8.81979615 | -7.14712243 | -6.33609215 |
| C | -8.93186055 | -7.37024473 | -7.71899846 |
| H | -9.52889049 | -6.73218397 | -8.35179867 |
| C | -8.23848436 | -8.43207116 | -8.28377135 |
| H | -8.29818508 | -8.58580932 | -9.35745505 |
| C | -7.44796075 | -9.28029466 | -7.49100879 |
| C | -7.41281280 | -8.98428243 | -6.11798487 |
| H | -6.84136434 | -9.58138593 | -5.41327476 |
| C | -10.16915263 | -4.90969646 | -6.25307323 |
| C | -10.53894857 | -3.95431351 | -5.10623765 |
| H | -9.65141989 | -3.61672700 | -4.56660492 |
| H | -11.03791615 | -3.08400479 | -5.54600723 |
| H | -11.22051934 | -4.42100498 | -4.39076038 |
| C | -11.46689527 | -5.35703222 | -6.95536712 |
| H | -12.12544630 | -5.87286732 | -6.24799260 |
| H | -11.99324407 | -4.46962419 | -7.32373851 |
| H | -11.30234496 | -6.01802752 | -7.80906020 |
| C | -9.18556505 | -4.18195180 | -7.19288938 |
| H | -8.95232013 | -4.74156944 | -8.10122531 |
| H | -9.63249652 | -3.22937931 | -7.49795425 |
| H | -8.24772961 | -3.97575048 | -6.66711845 |
| C | -6.68390554 | -10.41763098 | -8.04547384 |
| C | -7.15504474 | -11.14088345 | -9.15615444 |
| H | -8.11713925 | -10.89137268 | -9.59669397 |
| C | -6.43189757 | -12.21021732 | -9.67794332 |
| H | -6.84246980 | -12.77040700 | -10.51378619 |
| C | -5.20531075 | -12.60413316 | -9.11354138 |
| C | -4.73726088 | -11.88028060 | -8.00232419 |
| H | -3.77859755 | -12.13604943 | -7.55972458 |
| C | -5.45831939 | -10.81144786 | -7.47683055 |
| H | -5.04743072 | -10.26019440 | -6.63576946 |
| C | -4.43463023 | -13.74567485 | -9.66907431 |
| C | -4.36062109 | -13.96180248 | -11.05709512 |
| H | -4.85178013 | -13.26769074 | -11.73419963 |
| C | -3.63660237 | -15.03498664 | -11.57910519 |

| | | | |
|---|---|---|---|
| H | -3.58538647 | -15.17615208 | -12.65593750 |
| C | -2.96993005 | -15.91699018 | -10.72382775 |
| H | -2.40542069 | -16.75244464 | -11.12978767 |
| C | -3.03427112 | -15.71492067 | -9.34218194 |
| H | -2.52634458 | -16.39874415 | -8.66665649 |
| C | -3.75841262 | -14.64145657 | -8.82104130 |
| H | -3.82081108 | -14.51015870 | -7.74400473 |
| Br | -6.19797318 | -5.48071681 | -4.31214792 |
| O | -9.35247232 | -8.62808952 | -2.78592718 |
| N | -9.47267440 | -8.61488179 | -1.49993439 |
| N | -8.06809722 | -6.76585710 | -1.57019590 |
| C | -8.81978821 | -7.58086732 | -0.80168025 |
| C | -8.93184414 | -7.35776620 | 0.58123030 |
| H | -9.52886350 | -7.99584177 | 1.21402574 |
| C | -8.23846954 | -6.29594506 | 1.14601324 |
| H | -8.29816232 | -6.14222330 | 2.21970012 |
| C | -7.44795652 | -5.44770515 | 0.35325704 |
| C | -7.41281704 | -5.74369780 | -1.01977166 |
| H | -6.84137599 | -5.14658107 | -1.72447753 |
| C | -10.16914310 | -9.81828853 | -0.88471927 |
| C | -10.53893534 | -10.77365402 | -2.03157073 |
| H | -9.65140561 | -11.11123947 | -2.57120134 |
| H | -11.03791244 | -11.64396486 | -1.59181597 |
| H | -11.22049711 | -10.30694827 | -2.74704800 |
| C | -11.46688522 | -9.37095119 | -0.18242539 |
| H | -12.12543413 | -8.85511185 | -0.88979831 |
| H | -11.99323613 | -10.25835921 | 0.18594336 |
| H | -11.30233385 | -8.70995906 | 0.67127034 |
| C | -9.18555712 | -10.54604007 | 0.05509370 |
| H | -8.95231113 | -9.98642613 | 0.96343227 |
| H | -9.63249017 | -11.49861257 | 0.36015486 |
| H | -8.24772221 | -10.75224087 | -0.47067723 |
| C | -6.68390130 | -4.31037465 | 0.90773266 |
| C | -7.15503522 | -3.58714070 | 2.01842861 |
| H | -8.11712497 | -3.83666153 | 2.45897132 |
| C | -6.43188752 | -2.51781212 | 2.54022807 |
| H | -6.84245498 | -1.95763726 | 3.37608258 |
| C | -5.20530599 | -2.12388411 | 1.97582243 |
| C | -4.73726300 | -2.84771762 | 0.86459043 |
| H | -3.77860338 | -2.59193874 | 0.42198711 |
| C | -5.45832150 | -3.91654454 | 0.33908567 |
| H | -5.04743707 | -4.46778372 | -0.50198706 |
| C | -4.43462494 | -0.98234824 | 2.53136647 |
| C | -4.36060574 | -0.76624178 | 3.91939045 |
| H | -4.85175737 | -1.46036569 | 4.59648862 |
| C | -3.63658702 | 0.30693656 | 4.44141111 |
| H | -3.58536266 | 0.44808560 | 5.51824554 |
| C | -2.96992317 | 1.18895544 | 3.58614267 |
| H | -2.40541329 | 2.02440567 | 3.99211105 |
| C | -3.03427483 | 0.98690710 | 2.20449421 |
| H | -2.52635569 | 1.67074275 | 1.52897511 |
| C | -3.75841685 | -0.08655117 | 1.68334246 |
| H | -3.82082325 | -0.21783264 | 0.60630430 |

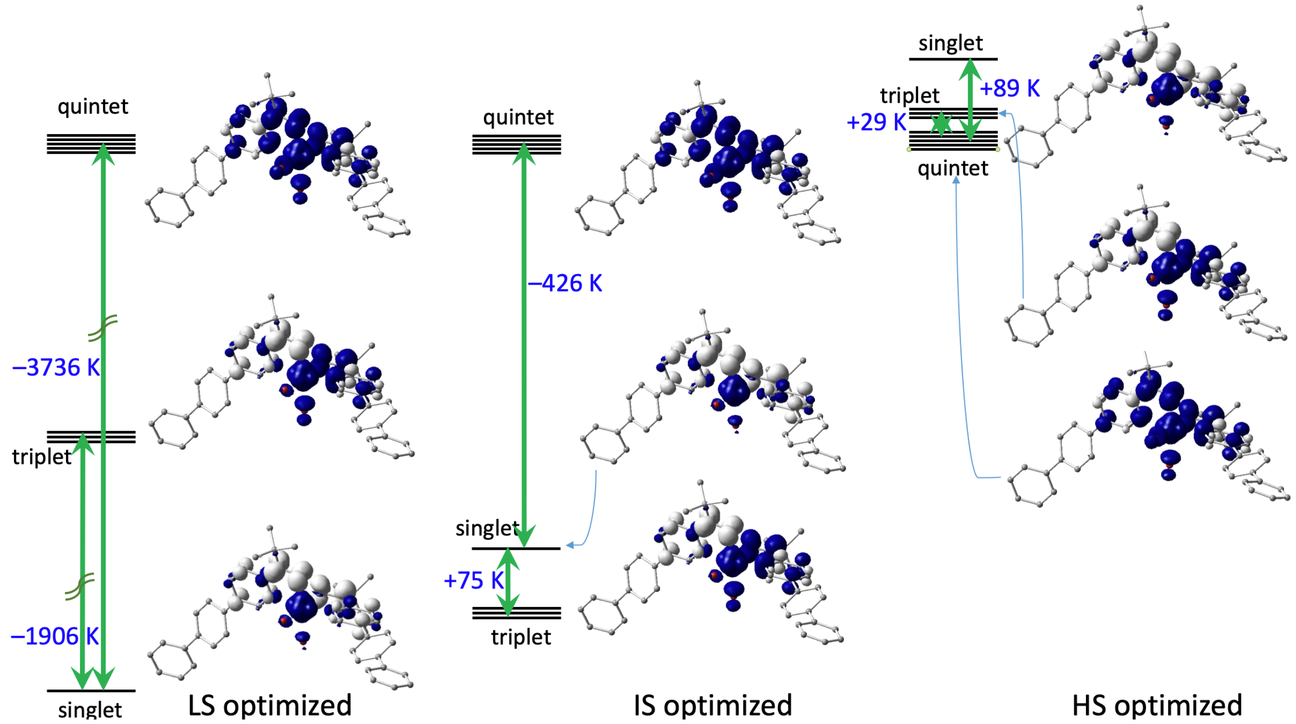


**Figure S10.** Energy level diagrams and spin density maps on the DFT optimized structures of [Ni(bppyNO)$_2$Br$_2$]. (left) On the structure optimized with the LS singlet state (the coordinates are given from **Figure S7**). (center) On the structure with the IS triplet state (from **Figure S8**). (right) On the structure with the HS quintet state (from **Figure S9**).

## References


S1. Stoll, S.; Schweiger, A. EasySpin, a comprehensive software package for spectral simulation and analysis in EPR. *J. Magn. Reson.* **2006**, *178*, 42-55.

S2. Gaussian 16, Revision C.01, M. J. Frisch, G. W. Trucks, H. B. Schlegel, G. E. Scuseria, M. A. Robb, J. R. Cheeseman, G. Scalmani, V. Barone, G. A. Petersson, H. Nakatsuji, X. Li, M. Caricato, A. V. Marenich, J. Bloino, B. G. Janesko, R. Gomperts, B. Mennucci, H. P. Hratchian, J. V. Ortiz, A. F. Izmaylov, J. L. Sonnenberg, D. Williams-Young, F. Ding, F. Lipparini, F. Egidi, J. Goings, B. Peng, A. Petrone, T. Henderson, D. Ranasinghe, V. G. Zakrzewski, J. Gao, N. Rega, G. Zheng, W. Liang, M. Hada, M. Ehara, K. Toyota, R. Fukuda, J. Hasegawa, M. Ishida, T. Nakajima, Y. Honda, O. Kitao, H. Nakai, T. Vreven, K. Throssell, J. A. Montgomery, Jr., J. E. Peralta, F. Ogliaro, M. J. Bearpark, J. J. Heyd, E. N. Brothers, K. N. Kudin, V. N. Staroverov, T. A. Keith, R. Kobayashi, J. Normand, K. Raghavachari, A. P. Rendell, J. C. Burant, S. S. Iyengar, J. Tomasi, M. Cossi, J. M. Millam, M. Klene, C. Adamo, R. Cammi, J. W. Ochterski, R. L. Martin, K. Morokuma, O. Farkas, J. B. Foresman, D. J. Fox, Gaussian, Inc., Wallingford CT, 2019.

S3. J. B. Foresman, Æ. Frisch, Exploring chemistry with electronic structure methods: A guide to using Gaussian. Gaussian Inc., Wallingford, CT, USA, 1996.
